# Spinal Nerves Schwannomas: Experience on 367 Cases—Historic Overview on How Clinical, Radiological, and Surgical Practices Have Changed over a Course of 60 Years

**DOI:** 10.1155/2017/3568359

**Published:** 2017-09-18

**Authors:** Jacopo Lenzi, Giulio Anichini, Alessandro Landi, Alfonso Piciocchi, Emiliano Passacantilli, Francesca Pedace, Roberto Delfini, Antonio Santoro

**Affiliations:** ^1^Department of Neurological Sciences, Neurosurgery, University of Rome “Sapienza”, Rome, Italy; ^2^Imperial College London, Imperial College Healthcare NHS Trust, Charing Cross Hospital, Department of Neuroscience, Neurosurgery, London, UK

## Abstract

**Background:**

Spinal schwannomas are common benign spinal tumors. Their treatment has significantly evolved over the years, and preserving neurological functions has become one of the main treatment goals together with tumor resection.

**Study Design and Aims:**

Retrospective review focused on clinical assessment, treatment techniques, and outcomes.

**Methods:**

A retrospective study on our surgical series was performed. Clinical and operative data were analyzed. In regard to neurophysiologic monitoring, patients were retrospectively divided into two groups comparing the outcomes before and after introduction of routine intraoperative neurophysiology tests.

**Results:**

From 1951 to 2010, 367 patients overall were treated. Diagnosis was obtained using angiography and/or myelography (pre-CT era), MRI, or CT scan. A posterior spinal approach was used for most patients; complex approaches were adopted for treatment of giant/dumbbell tumors. A trend of neurophysiology monitoring decreasing the rate of post-op neurological deficits was observed but was not statistically significant enough to draft evidence-based conclusions.

**Conclusions:**

Clinical and radiological assessment of spinal schwannomas has markedly changed over the course of 50 years. Diagnostic tools have improved, and detection of recurrence has become way more sensitive. Neurophysiologic monitoring has become a useful intraoperative tool to guide resection and prevent post-op neurological impairment.

## 1. Introduction

Solitary spinal nerve schwannomas or neurinomas are the most common nerve sheath tumors of the spine [1–4]. A male-to-female ratio of 1 : 1 has been reported, although slight prevalence of males has been recently noted in one of the largest series [5]; clinical presentation is most common during the fourth and fifth decades of life [6, 7]. These tumors typically arise from Schwann cells of a sensory nerve root; they appear as a globular, well-defined, encapsulated mass, well defined and separated from the other rootlets [8]. Gold standard treatment for symptomatic spinal schwannomas is complete surgical resection, which stops symptoms progression, helps recovery in most patients, and decreases the rate of recurrence. Radiotherapy can be considered as second-choice treatment in patients who are not good candidates for surgery, or for recurrent tumors [9–12]. Recent findings suggest that Ki-67 index might be related to the likelihood of recurrence when a residual is left behind [13].

Surgical removal using a standard midline posterior approach is feasible in most patients. Total removal of the mass is performed by isolating the tumor from the surrounding nervous structures (spinal cord and/or nerve roots) and then carefully dissecting it from afferent nerve root. Though debated, cutting nerve root is a relatively common choice during schwannomas surgery. Bearing in mind that sensory root origin is way more common and that functional compensation by surrounding spinal roots has been demonstrated [14–16], most authors report that cutting nerve root does not significantly increase the risk of postoperative neurological deficits [17–19]. Furthermore, the rate of recurrence is likely to be higher when the spinal root is preserved, although data about this topic are conflicting. Development of intraoperative neurophysiologic monitoring over the last 15 years has altered this point of view. Despite being rare, schwannomas originating from motor roots are reported almost in every surgical series, and in some cases severe postoperative motor deficit was observed when the nerve was cut [20]. Debate about this point is ongoing, as today's goal of surgical treatment is not anymore considered just tumor removal, but also preserving the patient's quality of life.

This study reports on an extended retrospective series of patients with diagnosis of spinal schwannoma treated in our institute over a 60 years' period. Clinical and radiological findings, preoperative evaluation, surgical technique, and neurophysiologic monitoring are described and discussed. Comparison between the premonitoring and the postmonitoring era results is reported and extensively analyzed.

## 2. Materials and Methods

From 1951 to 2010, a total number of 590 patients with diagnosis of spinal nerve tumors were treated in our department. Only patients with histological diagnoses of schwannoma were considered. Neurofibromas and malignant peripheral nerve sheath tumor were excluded. The following parameters were considered: sex, age, clinical and radiological presentation (including level and extension of the tumor), surgical approach, histology, and clinical and radiological follow-up. All patients with clinical and molecular diagnoses of neurofibromatosis (NF-1) and the single patient with multiple schwannomata in the absence of neurofibromatosis (schwannomatosis) [21] were excluded. Follow-up lasted from one to twenty years after surgery; average time was 5-6 years; median was 10 years; twenty-three patients were lost to follow-up and were excluded by the present study, so 367 patients overall were considered.

### 2.1. Clinical Evaluation

From 1951 to 1991, all clinical data were obtained from the patients' medical records. Only medical records with complete preoperative and postoperative neurological evaluations were considered. The following clinical data were recorded: pain, grade of sensitive and motor deficit (due to root and/or medulla compression), and presence of sphincter disturbance.

From 1991 to 2009, all patients were assessed with neurological evaluation. All patients with clinical signs of spinal root deficit also underwent pre- and postoperative neurophysiological studies (see Neurophysiologic Evaluation).

### 2.2. Radiological Assessment

At least one preoperative radiological exam was performed, and patients were followed up with a series of postoperative clinical and radiological exams.

Before the CT era, from 1951 to 1976, patients were studied with preoperative myelography and/or spinal angiography. In this specific group of patients, follow-up was mostly performed by clinical examination. Postoperative radiological evaluation was performed only in patients with strong clinical indications of recurrence.

CT scans with contrast were performed on all patients before the advent of MRI, from 1976 to 1987, and in another three additional patients who could not undergo MRI due to the presence of an incompatible device (e.g., pacemaker). In this group of patients, CT scan was used also for follow-up.

MRI was performed on all remaining patients, both for preoperative evaluation and for postoperative follow-up. Postoperative CT scans or MRI scans were performed following surgery, six months postoperatively, and then yearly. Patients without any evidence of recurrence after five years of follow-up were considered free of disease.

### 2.3. Neurophysiologic Evaluation

In the last twenty years, all patients with clinical indications of spinal nerve root deficits were evaluated with neurophysiologic studies. Specifically, preoperative electromyography and nerve conduction studies were performed on patients with signs of spinal root motor and/or sensory deficits in order to assess the degree of nerve root impairment. Postoperative electromyography was performed only on patients with residual deficits after six months.

From 1991, intraoperative neurophysiologic monitoring (NPhM) was performed in all patients, but with different techniques depending on locations:

- Cervical spine schwannomas: SEP, MEP (in selected cases), EMG, and nerve conduction study
- Thoracic spine: SEP and MEP (in selected cases)
- Lumbar spine: EMG and nerve conduction study

Electrodes used for SEP recordings and MEP stimulation were positioned according to the International 10–20 system [22].

SEP and MEP were performed in call cases where spinal cord compression was seen on radiological investigations. SEP were recorded to evaluate functional integrity of posterior column/medial lemniscus sensory ascending pathways. SEP from legs were recorded using stimulation on the tibial nerve near the malleolus and recordings on the head from two points located 2 cm posterior to* Cz* and* Fz*. Tibial nerve was stimulated from both sides using plate or needle electrodes; stimulation was 0.2 msec in length and intensity was set after appearance of muscle contractions. Electrodes impedance was under 3 Ω; potentials were filtered and amplified with filters set at 3.00 Hz. Signals mean result was obtained by using an average of 200 measurements for every recording and then comparing it to the basal signal; length of the analysis was 30–50 msec. SEP from the upper arms were recorded on the scalp, 2 cm posterior to* C3* and* C4*, after stimulation of the median nerve. A reduction of the signal range under 50% during surgery was considered significant for posterior column/medial lemniscus sensory ascending pathways distress.

MEP were used to evaluate functional integrity of the cortical-spinal tract in patients with cervical and thoracic schwannomas. Electrodes were positioned on the scalp using* C1*,* C2*,* C3*, and* C4*. Five to 7 electrical stimulations were administered on the scalp, using the following parameters for every stimulation: 200 mA intensity, 0.5 msec length, and 2 to 4 msec gap between each stimulation. Muscle response recordings were obtained with needle electrodes; abductor pollicis on both arms and the anterior tibial muscle on both legs were selected for this purpose. Seriated stimulations were performed under general anesthesia. During surgery, presence or absence of MEP was constantly evaluated. EMG and nerve conduction tests were not used in patients with schwannomas of the thoracic tract, as nerve cutting was considered to cause minimal effects on the quality of life of these patients.

Patients with diagnosis of cervical and lumbar tract/cauda equina schwannomas were monitored with intraoperative EMG and nerve conduction studies. Both spontaneous activity and cMAP were evaluated for this purpose. During cervical roots monitoring, bilateral electrodes were positioned on biceps, triceps, extensor carpi, and abductor pollicis; lumbar roots monitoring was obtained with bilateral electrodes positioned on quadriceps, anterior tibialis, triceps surae, and sphincter ani externus. Spinal roots were stimulated using a bipolar stimulator with impulse administered directly on the root. Positive response was considered as strongly suggestive of a functionally active nerve root when obtained with the following settings: ≤0.3 up to 0.6 mA intensity, 0.05 msec length, and 200 msec latency. Presence, spread, and possible reduction/disappearance of cMAP were constantly evaluated during tumor resection, together with spontaneous muscle activity. Presence of sudden, high-frequency electric shots on the muscles monitored was considered a sign of severe spinal root stretching, traction, or damage during tumor resection, while low-frequency shots were interpreted as a consequence of a mild and temporary spinal root sufferance.

### 2.4. Surgical Approaches

All patients included received surgery as first-line treatment. A posterior approach with posterior laminectomy was performed in most cases, while in the last few years posterior laminotomy was used in some of them (see below). Combined or anterior approaches were used in selected cases. Operative microscope and microsurgical instrumentation were introduced in 1970 in our department: from that year onwards, all patients were operated on under microscope magnification. Before the operative microscope era, all our surgeons used to operate these tumors with the aid of surgical loupes.

Schwannomas of the craniocervical junction and high cervical region (C0–C2) extending anteriorly were treated with a posterior approach with suboccipital extension (when required) or with a posterior-lateral (far lateral) approach [23–26]. Dumbbell tumors of the cervical tract (C3–C6) with extracanalar extension were treated with a combined posterior and anterior approach. Anterior access to the cervical tract and cervical foramina was obtained using George's approach [27, 28].

Schwannomas of the thoracic tract with extracanalar extension were approached considering thoracic extension and proximity of the tumor to vascular structures. Most of these cases were treated through a transpedicular approach or costotransversectomy. Selected tumors showing anterior intrathoracic extension and proximity to vital vessels (aorta and/or vena cava) were treated with a lateral extracavitary approach or a combined posterior and transthoracic approach.

Schwannomas of the lumbar region were treated with posterior laminectomy/laminotomy, extended with facetectomy, or even a transpeduncular approach in selected patients with dumbbell tumors.

## 3. Results

The present study included 367 patients overall, 189 males and 178 females (M : F = 1), with a mean age of 43 years and a range from 4 to 81 years. Follow-up lasted from one to twenty years after surgery; mean was 4–6 years; median was 10 years. Clinical presentations are depicted in Table 1. The most common symptom was pain, followed by signs of spinal root deficits, pyramidal tract compression, and ultimately sphincter disorders.

As expected, symptoms were mostly related to location of the mass. Lumbar tract was the most common site of origin, as also seen in most literature (see Table 2) [2]. Tumors of the thoracic tract were also common, followed in occurrence by cervical tract. Craniocervical, cervicothoracic, and thoracolumbar junctions were rare but possible locations.

From 1951 to 1975, 153 patients underwent preoperative evaluation with spinal myelography and/or angiography. Three patients showing clinical suggestion of recurrence underwent angiography at follow-up. Since 1976, a total of 214 patients underwent pre- and postoperative evaluation with CT or MRI with contrast.

As mentioned previously, posterior laminectomy/laminotomy was the preferred approach. Laminectomy was used in 313 patients, while laminotomy was recently introduced in our department and used in 16 patients; five of them were reported in a previous series [29].

**Figure 1:**
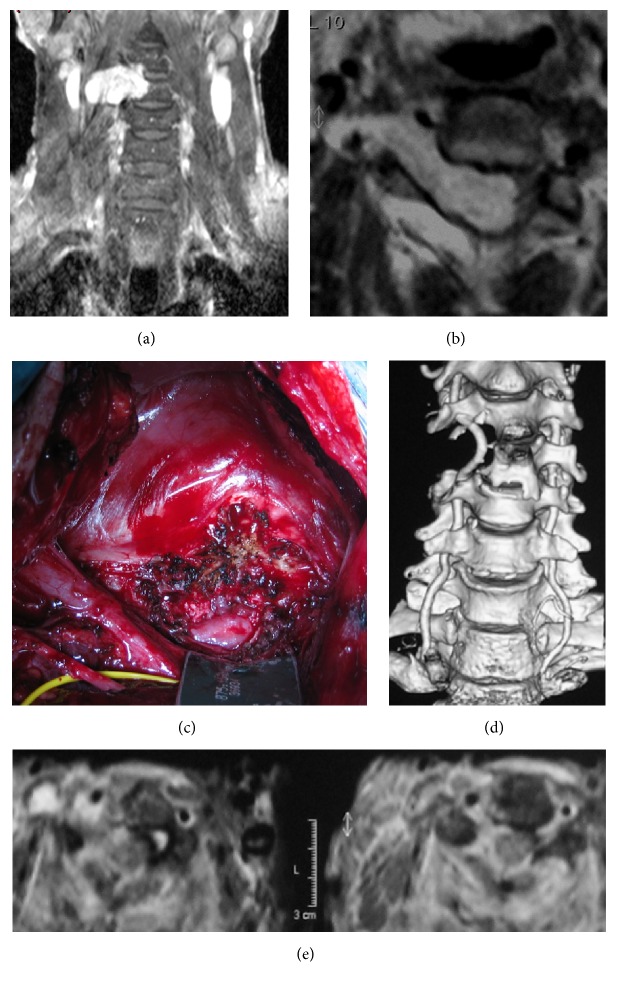
Illustrative case. A 58-year-old man presenting with right arm weakness, paraparesis, and increased reflexes: (a) T1w MRI with contrast, coronal view showing dumbbell schwannoma at C3-C4 level with bone erosion of the vertebral body; (b) T1w MRI, axial section; vertebral artery was anteriorly dislocated (white arrow) and combined, two-step anterior-lateral and posterior approaches to the cervical spine were performed; (c) intraoperative view during anterior-lateral approach; (d) postoperative CT scan showing surgical excision of the tumor with preservation of the vertebral artery; (e) T2w postoperative MRI, axial view.

In all remaining patients, surgical approaches were chosen considering the extracanalar extension of the schwannoma. In cases involving craniocervical junction and high cervical tract (C0–C2), 11 patients were treated with a posterior approach, with suboccipital extension in 2 cases, and the remaining 8 patients were treated with a posterior-lateral (far lateral) approach. Five dumbbell tumors of the cervical tract (various locations from C3 to C7) were resected using an anterior-lateral approach [8] (Figure 1). Dumbbell neurinomas of the thoracic tract were treated in 7 cases with a transpeduncular approach and in 4 cases with a costotransversectomy. A single patient with a giant schwannoma located near the aorta required a combined posterior and transthoracic approach. Three patients (two lumbar, one thoracic location) with giant, dumbbell-shaped schwannomas required a transpeduncular approach with further stabilization due to preoperative instability observed with MRI and X-ray.

In regard to the neurophysiologic assessment, patients were retrospectively divided into two main groups: group A including 226 patients treated before the NPhM era (1951–1991) and group B including 141 patients treated with the assistance of NPhM (1991–2010). A sensory deficit with intact gross sensory function (touch with or without pain and/or heat) and/or sensory function preserved but with paresthesias/dysesthesias were both considered as partial sensory deficits; cases with complete anesthesia or with paresthesias/dysesthesias without any gross sensation preserved were considered as complete loss of sensory function. Motor function loss was considered as partial in cases where motor strength was 3/5 or more, while it was considered as complete/severe when motor strength was below 2/5 (see Table 1). Before the introduction of NPhM, nerve root at the origin of the tumor was sacrificed in 199 cases (88%). Out of this group, 55 patients showed postoperative sensory deficits, transient in only 13 of these cases, while complete anesthesia was noted in the remaining 42; out of this group, 34 patients had thoracic schwannomas. Twenty-seven patients showed clinically evident postoperative motor root deficit, due to motor root cutting, and 20 of them showed partial improvement (more than 3/5 motor strength) at follow-up. Seven patients showed persisting and complete or severe (0–2/5) motor deficit: C5 and C6 deficit (no extension of wrist and elbow) in one case, isolated C5 deficit in 2 cases (poor wrist extension), L4 deficit in 1 case (impaired walk due to loss of ankle dorsiflexor), L5 deficit in 2 cases (impaired walk due to loss of extension of the foot, complete in one case), and S1 deficit in one case (impaired walk due to complete loss of foot flexion). After introduction of NPhM, nerve root was sacrificed in 89 cases (63%). Out of this group, only 15 cases showed positive intraoperative stimulation, although only in one of them the response was considered significantly positive (= still positive below 3 mA). No postoperative motor deficit was noted in any of these cases except one. All roots sacrificed were sensory, and in 6 cases postoperative focal anesthesia persisted at follow-up. Functionally significant motor root was sacrificed in one patient due to the presence of a large thrice-recurring intraextradural tumor; this patient showed complete (0/5) C6 motor deficit postoperatively, with no possibility of wrist extension, and partial motor deficit on motor nerve roots nearby (C5 and C7), which recovered at follow-up. Comparing group A and group B, the presence of NPhM did significantly decrease incidence of postoperative motor root deficit in early postoperative period (*p* = 0.06) but did not at follow-up (*p* = 0.13) (Table 3).

Regarding spinal cord-related signs, the great majority of patients showed full or partial recovery of preoperative symptoms and/or signs. Root pain resolved in nearly all cases, only persisting in 9 patients, while back pain persisted in a higher percentage of patients (Table 1). Pyramidal and root deficits showed a slower but significant recovery. Sphincter compromise presented the worst prognosis: only a small percentage of cases showed a full regression of preoperative signs.

Gross total resection was achieved in all but 12 patients (Table 4). In all these cases, the surgeon reported a difficult resection due to presence of a very strict tumor adherence or infiltration to the root affected and/or the strong evidence of a functionally important nerve root, not suitable for safe cutting. Out of this group, 9 patients showed giant tumors, intra- and extradural, with extension outside the spinal canal. Seven patients belong to group A, while just 2 patients belong to group B. The residual tumor was controlled at follow-up with angiography and/or myelography in 3 patients, CT scan in 1 patient, and MRI in 5 patients. Five patients of this group showed tumor recurrence, one after 6 months and then 2 years after another surgical resection, one after 15 month, and 3 after 3 years. The remaining 4 patients showed no growth of the residual tumor at follow-up.

In the remaining four patients with partial resection, tumor showed strict adherence to a nerve root, so it was decided to leave a small amount of it without cutting/stretching the nerve root. All these patients belong to group B. The small residual was controlled at follow-up with seriated MRI. One of them showed tumor recurrence after 2 years, one after 15 months (see below); in the remaining 2 patients, the residual was stable at follow-up.

Twenty-two patients overall showed tumor recurrence, seven of them after incomplete resection (Table 4). Recurrence was evaluated considering the different surgical strategy between group A and group B; only the 15 patients with gross total resection were considered for this purpose. Nine patients with tumor recurrence belonged to group A, while 6 patients were operated on after introduction of NPhM (group B). Nine patients of group A were operated on by cutting the root of origin in 3 cases and preserving it in the remaining 6. All patients of group B were treated without cutting of the nerve root.

Morbidity was observed as follows: 28 cases showed persistent CSF leakage; 14 patients developed wound infections, all but one successfully treated with surgical debridement and antibiotic therapy; 4 cases developed delayed kyphosis requiring surgical realignment and fusion. Mortality rate was less than 1%: 1 patient died for PE on 3rd post-op day, and 1 patient with wound infection developed meningitis, which eventually led to respiratory and cardiac arrest.

## 4. Discussion

### 4.1. Epidemiology

Solitary or syndromic spinal nerve schwannomas are considered the most common primary spinal tumors [2–5, 20]. Many clinical and surgical series are reported in the literature, showing relatively uniform data about prevalence and incidence.

Males and females are equally affected, and the age of onset is usually between 25 and 50 years [4, 20]. Hirano and colleagues reported an extended series of 678 spinal cord tumors: schwannomas were the most common histological type, with a slight prevalence of male sex (M : F = 1.3 : 1) and onset between 50 and 59 years of age [5]. Symptoms are related to tumor location and its proximity to spinal cord and nerve roots. As in our series, most authors report pain as the onset symptom, followed by sensory deficits (mostly paresthesias) [2, 6]. Motor deficits and sphincter impairment are uncommon onset symptoms. Without the essential aid of neuroimaging, diagnosis can only be presumed.

### 4.2. Radiological Assessment and Location

**Figure 2:**
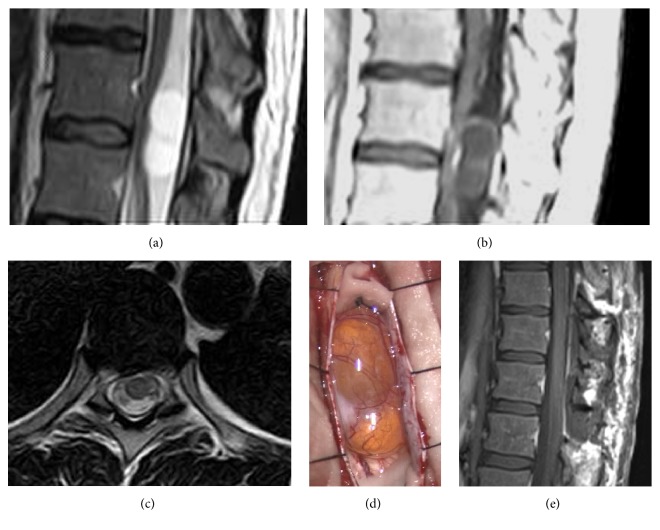
Illustrative case. A 43-year-old woman came to our observation complaining from pain on the left chest with irradiation to the groin. Neurological examination showed slightly increased reflexes: (a) MRI T2w sequences, sagittal view, showing a posterior mass with marked cystic degeneration; (b) T1w sequences showing poor peripheral contrast enhancement; (c) T2w sequences, axial view; (d) intraoperative photography showing a neurinoma with marked cystic degeneration; (e) postoperative MRI, T1w sequences, sagittal view.

At the dawn of the microsurgery era, tests available to confirm diagnosis were angiography and myelography. These procedures only allowed for detection of the mass, without any clear clue regarding its relationship with surrounding vascular and nervous structures. Introduction of CT scan had a major impact on the diagnostic process. However, before the introduction of MRI, follow-up of these lesions was extremely difficult; many patients with small spinal schwannomas were misdiagnosed, and so was tumor residual/recurrence. Today, MRI is the gold standard for preoperative diagnosis of spinal schwannoma. It clearly shows that these tumors usually are rounded-shaped and well defined, showing homogeneous enhancement after gadolinium administration. Occasionally, cystic degeneration can be noted and it can decrease contrast diffusion (see Figure 2). In selected cases of giant tumors, adding CT scan might help to evaluate the degree of bone erosion and potential need for spinal stabilization.

Schwannomas are more commonly seen in the lumbar tract. As reported by Jinnai et al., anatomic features of lumbar nerve roots, which run long distances from the conus to the foramina, could probably explain the higher percentage of lumbar schwannomas and why the incidence of intraextradural tumors decreases as you move from the cervical to the lumbar spine [2]. Rarely, schwannomas arising from or strictly adherent to conus medullaris have been reported [2, 30].

### 4.3. Surgical Technique

Surgical resection is considered the gold standard for treatment of spinal schwannomas. It shows advantages in terms of both relieving mass effect and getting a diagnosis. Some authors reported good results with radiosurgery in selected cases (i.e., tumor recurrence, incomplete resection, or surgery not feasible due to comorbidities) [31]. However, radiotherapy can be considered as second-choice treatment in cases where there is no indication for surgery or for recurrent tumors [9–11]. Radiotherapy shows short-term control of the disease, although long-term results and rate of complications are still a matter of debate [12]. Factors influencing recurrence are size of the lesion and degree of resection, while location does not seem to play a role [32, 33].

Surgical treatment of spinal schwannomas has not changed its basic principles among years and decades. Traditional posterior approach through laminectomy/laminotomy is still the preferred surgical strategy (Figure 2), while combined and/or invasive approaches are limited to dumbbell-shaped tumors with large extraspinal components [2, 6, 33]. Opinions regarding the ideal surgical approach for complex schwannomas with extradural extension are heterogeneous. Asazuma et al. proposed an imaging-based radiological classification of dumbbell tumors, analyzing the anatomic relationships of the tumor with surrounding anatomic structures; as in our series, the authors concluded that extensive and/or combined approaches are only required in those tumors with massive extraspinal extension [34]. Moreover, even in those cases, balance of risks and benefits of a lateral approach should be assessed on the specific case; as McCormick et al. pointed out, dumbbell schwannomas of the cervical spine with limited foraminal or also extraforaminal component can be safely removed through unilateral facet joint removal [35]. The same observations have been reported by Banczerowski et al., using a hemisemilaminectomy combined with partial lateral facet joint removal [8].

In our series, as in almost all the series reported, a posterior approach with laminectomy was used for the great majority of our patients (329 overall) and only dumbbell tumors with massive extracanalar extension required more complex or combined surgical approaches (see Figure 1).

Up until 2009, almost all patients were treated with a posterior laminectomy, associated with arterectomy and even partial pedicle resection when needed. From 2009 onwards, and just in selected cases, laminotomies were performed with reconstruction after surgical removal of the tumor [29].

After 1970, operative microscope was introduced in our department, thus playing a significant role in evolution of surgical resections of spinal schwannomas; before that time, operative loops were used. It is reasonable to suppose that this difference has a significant statistical weight, but we miss acceptably homogeneous data to conduct a multivariate analysis (missed mention on the operative record).

### 4.4. Introduction of NPhM

**Figure 3:**
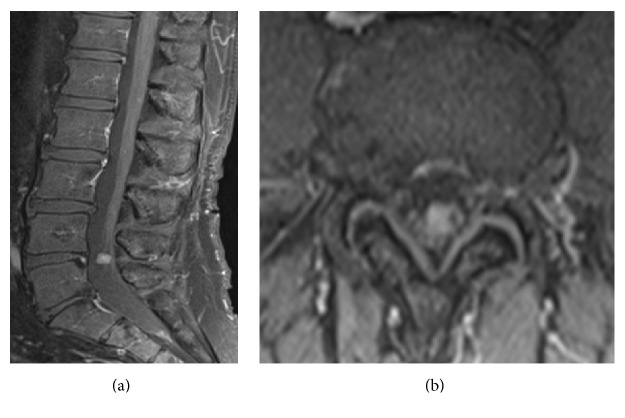
Illustrative case. A 61-year-old man presenting with anesthesia on the anterior portion of the right leg. (a) Preoperative MRI, sagittal sequence; (b) preoperative MRI, axial sequence. The patient underwent surgical intervention with NPhM; the schwannoma was found to have strict adherence with a sensitive root and to arise from a motor root; intraoperative stimulation showed high activation pattern of L4 (see video). Schwannoma was removed carefully and preservation of the nerve root was obtained. Postoperative course was uneventful.

Pre- and intraoperative neurophysiologic evaluation has now become routinely used to assess the degree of neurological damage and determine the functional role of a nerve root. Most spinal schwannomas arise from a sensory rootlet [36, 37]. Nerve roots of origin and their vascular supply seem to be completely free of pathological features upon microscopy examination, although axons of nerve roots are abundant in both the tumor capsule and tissue (Hasegawa, 2001). It has been suggested that the affected root slowly deactivates into a functionally neuroapraxic state, and its functions are progressively compensated by nearby roots [14–16]. These findings could explain why postoperative neurological deficits are limited or nonexistent also when the root of origin is cut. Moreover, as previously reported, a fair number of patients with postoperative deficits showed partial or complete recovery after a number of months or years [19, 38]. With that said, schwannomas affecting motor roots are indeed described almost in every surgical series, and in some cases severe postoperative motor deficit was observed (see illustrative case, Figure 3, and video). Today, the goal of surgical treatment cannot be considered just the removal of the tumor, but also preserving the patient's quality of life.

In analyzing our institute's past practice before application of NPhM (1951–1990), we noticed how nerve root of origin was always cut in order to prevent risk of tumor recurrence. However, compared with a relatively low number of recurrences (9 overall), seven cases of complete (0/5 motor strength) postoperative deficit (3%) were observed and 42 patients were observed with persistent complete sensory loss (18%). Introduction of NPhM gradually changed practice and preoperative planning. Since 1991, we started removing tumors while preserving functionally important nerve root as much as possible. This was extremely useful in identifying the few cases involving motor roots (see illustrative case). NPhM was also helpful in identifying the root of origin from other surrounding roots: in some patients, a single bundle can be adhered to the tumor; it can be mistaken for the root of origin and then cut with disastrous effects. Our results confirm that, after introduction of NPhM, incidence of postoperative neurological deficits markedly decreased. Only in one patient (0.7%) was the motor root cut, though intentionally. This was due to a giant schwannoma in its third recurrence. Before proceeding with surgery, the patient was extensively informed about his condition and the possibility of severe postoperative deficit. In all remaining cases showing positive response at intraoperative stimulation, nerve root was preserved at the cost of leaving a small amount of tumor (Figure 3). Once the nerve stimulation gave positive motor activation, complete surgical resection was performed using blunt dissection of the tumor from the root, trying as much as possible to preserve root integrity and not stretch it during dissection maneuvers (see video). As reported in the Results, the residual was left mainly in patients showing strict adherence of the tumor to the root, without a clear cleavage plane useful for complete resection.

Data regarding impact of NPhM on extent of resection and postoperative neurological status are still not convincing from a statistical perspective. While a positive trend could be detected, observations are still not solid enough to draft definitive conclusions. Interestingly, some authors reporting more recent series and pool of data also failed to show a statistically significant advantage of using NPhM in terms of neurological complications but had definitively positive results in terms of degree of tumor removal [20]. While we did not observe a significant difference in extent of resection between group A and group B (Table 4), analysis on the amount of resection in relationship with NPhM was not considered reliable in our series. This was due to nonhomogeneous data taken from the operative records. With that said, we would strongly recommend use of NPhM in schwannoma surgery, both for clinical and for medicolegal reasons. When informally interviewed about this point, most surgeons from our department advocated NPhM as a required tool in spinal schwannoma surgery. It is questionable whether it would be ethically appropriate to prospectively randomize patients undergoing schwannoma surgery in regard to NPhM, but more solid, statistically validated data are definitively desirable.

### 4.5. Limitations

This study has all the limitations of a long-term retrospective series. Data collected were standardized and analyzed as homogeneously as technically feasible. However, neurological evaluation was conducted by at least 8 different teams over the years, using different scales and methods. Radiological assessment changed from invasive myelographic examinations to modern MRI scans. Diagnosis and management gradually became quicker and more effective, due to both progressively increasing epidemiological data on schwannomas' natural history and the availability of new diagnostic techniques. The number of patients coming to our observation followed a relatively constant trend without a significant increase, but we presume this is mainly due to opening of new neurosurgical units on the same catching area. Data extrapolated from operative records were at times not homogeneous and incomplete, which is one of the main limitations of this retrospective review.

In summary, changes of practice in this field from 1950 and 2010 have been massive, and hence the improvement in diagnosis and treatment, which explain the difficulties of standardizing the set of data available. This is also the main reason why a proper multivariate analysis on the role of NPhM was not conducted. In this perspective, observations regarding the risk of recurrence in relationship with preservation of the nerve root should be interpreted with extreme caution, due to lack of statistically validated data.

## 5. Conclusions

Despite ground-breaking advancement in assessment and diagnosis, the surgical technique for resection of spinal schwannomas has not significantly changed over years; in the great majority of cases, a posterior laminectomy/laminoplasty is the preferred surgical access. Complete surgical resection should always be the aim of surgery, and it is feasible in the great majority of patients without any residual postoperative deficit. Proper preoperative and intraoperative neurophysiologic monitoring should be considered in order to identify those rare cases of schwannomas originating from a functionally active nerve root; when possible, cutting a motor root should be avoided in order to preserve the patient's quality of life.

## Supplementary Material

Intraoperative stimulation showing high activation of the stimulated root at the minimal possible setting - 0.3 mA in intensity.

## Figures and Tables

**Table 1:** Onset symptoms and postoperative course. In 199 cases of group A, root of origin was sacrificed. In this group, we found a higher percentage of complete postoperative sensitivity (18%) and motor (3%) deficits. In patients of group B, nerve root was almost always preserved when a positive intraoperative response was seen, and postoperative morbidity significantly decreased.

	1951–1991: 226 patients										
	Nerve root disturbance				Myelopathy				Sphincteric disturbance: 39 pts (17%)	Pain: 124 pts (55%)	
	Motor: 54 pts (24%)		Sensory: 84 pts (37%)		Motor: 27 pts (12%)		Sensory: 24 pts (11%)				
	*Partial*	*Complete/severe*	*Partial*	*Complete/severe*	*Partial*	*Complete/severe*	*Partial*	*Complete/severe*		*Root pain*	*Back pain*

Preoperative	54 (24%)	0	65 (29%)	19 (8%)	27 (12%)	0	23 (10%)	1 (0,4%)	39 (17%)	37 (16%)	87 (62%)
Postoperative											
One month	21 (9%)	9 (4%)	13 (6%)	55 (24%)	25 (11%)	0	16 (7%)	1 (0,4%)	28 (12%)	6 (3%)	73 (32%)
Six months	20 (9%)	7 (3%)	10 (4%)	42 (18%)	12 (5%)	0	5 (2%)	1 (0,4%)	28 (12%)	6 (3%)	26 (11%)
One year	20 (9%)	7 (3%)	10 (4%)	42 (18%)	7 (3%)	0	3 (1%)	1 (0,4%)	28 (12%)	6 (3%)	21 (9%)
Five years	20 (9%)	7 (3%)	10 (4%)	42 (18%)	7 (3%)	0	3 (1%)	1 (0,4%)	28 (12%)	6 (3%)	21 (9%)

	1991–2009: 141 patients										
	Nerve root disturbance				Myelopathy				Sphincteric disturbance: 11 pts (8%)	Pain: 85 pts (60%)	
	Motor: 25 pts (18%)		Sensory: 51 pts (36%)		Motor: 20 pts (14%)		Sensory: 13 pts (9%)				
	*Partial*	*Complete/severe*	*Partial*	*Complete/severe *	*Partial*	*Complete/severe*	*Partial*	*Complete/severe*		*Root pain*	*Back pain*

Preoperative	25 (18%)	0	48 (34%)	3 (2%)	20 (14%)	0	13 (9%)	0	11 (8%)	26 (31%)	59 (69%)
Postoperative											
One month	12 (8%)	1 (0.7%)	17 (12%)	8 (11%)	18 (12%)	0	7 (5%)	0	6 (4%)	4 (3%)	41 (29%)
Six months	9 (6%)	1 (0.7%)	6 (9%)	6 (8%)	11 (8%)	0	5 (3%)	0	6 (4%)	3 (2%)	19 (13%)
One year	8 (6%)	1 (0.7%)	4 (3%)	6 (8%)	5 (3%)	0	4 (3%)	0	6 (4%)	3 (2%)	12 (8%)
Five years	8 (6%)	1 (0.7%)	4 (3%)	6 (8%)	5 (3%)	0	4 (3%)	0	6 (4%)	3 (2%)	12 (8%)

**Table 2:** Sites of origin and surgical approaches used for our patients.

Sites	Number of patients	Surgical approach					
		Posterior laminectomy/laminotomy	Far lateral approach to CCJ	Anterior lateral approach to cervical region	Transpedicular approach	Costotransversectomy	Transthoracic
Craniocervical junction (C0–C2)	19	0	19	0	0	0	0
Cervical tract (C3–C7)	85	79	0	6	0	0	0
Cervicothoracic junction (C7-T1)	20	18	0	0	0	0	2
Thoracic tract (T1–T12)	106	97	0	0	5	4	1
Thoracolumbar junction (T12-L1)	23	21	0	0	2	0	0
Lumbar tract (L1-S3)	114	114	0	0	0	0	0

Total	367	329	19	5	7	4	3

**Table 3:** Postoperative complete/severe motor nerve root disturbance: comparison between group A and group B.

	1951–1991	1991–2009	*p* value
Postoperative motor nerve root disturbance, complete/severe (%)			
One month	4.0	0.7	0.06
Six months	3.1	0.7	0.13
One year	3.1	0.7	0.13
Five years	3.1	0.7	0.13

**Table 4:** Tumor recurrence related to partial or complete surgical removal. No significant difference between recurrence percentages in group A and group B was noted. However, data were not statistically strong enough to draft definitive conclusions.

	Tumor recurrence (22 pts)			
	Partial removal (7 pts)		Gross total resection (15 pts)	
	Giant tumor	Nerve root strict adherence	Nerve root sacrificed	Nerve root preserved
Group A	4	0	3	6
Group B	1	2	0	6

## Abbreviations

NPhM: Neurophysiological monitoring
CSF: Cerebrospinal fluid
MEP: Motor evoked potential
SEP: Sensory evoked potential
EMG: Electromyography
cMAP: Compound motor action potential
Ω: Ohm
CT: Computed tomography
MRI: Magnetic resonance imaging
PE: Pulmonary embolism.
